# The Influence of Weather Variation, Urban Design and Built Environment on Objectively Measured Sedentary Behaviour in Children

**DOI:** 10.3934/publichealth.2016.4.663

**Published:** 2016-09-01

**Authors:** Tarun Reddy Katapally, Daniel Rainham, Nazeem Muhajarine

**Affiliations:** 1 Johnson Shoyama Graduate School of Public Policy, University of Regina, Regina, Canada; 2 Department of Community Health and Epidemiology, College of Medicine, University of Saskatchewan, Saskatoon, Canada; 3 Healthy Populations Institute, Dalhousie University, Halifax, Canada; 4 Saskatchewan Population Health and Evaluation Research Unit, University of Saskatchewan, Saskatoon, Canada

**Keywords:** sedentary behaviour, physical inactivity, children, urban design, built environment, neighbourhoods, active living research, weather, weather variation, accelerometry

## Abstract

With emerging evidence indicating that independent of physical activity, sedentary behaviour (SB) can be detrimental to health, researchers are increasingly aiming to understand the influence of multiple contexts such as urban design and built environment on SB. However, weather variation, a factor that continuously interacts with all other environmental variables, has been consistently underexplored. This study investigated the influence of diverse environmental exposures (including weather variation, urban design and built environment) on SB in children. This cross-sectional observational study is part of an active living research initiative set in the Canadian prairie city of Saskatoon. Saskatoon's neighbourhoods were classified based on urban street design into grid-pattern, fractured grid-pattern and curvilinear types of neighbourhoods. Diverse environmental exposures were measured including, neighbourhood built environment, and neighbourhood and household socioeconomic environment. Actical accelerometers were deployed between April and June 2010 (spring-summer) to derive SB of 331 10–14 year old children in 25 one week cycles. Each cycle of accelerometry was conducted on a different cohort of children within the total sample. Accelerometer data were matched with localized weather patterns derived from Environment Canada weather data. Multilevel modeling using Hierarchical Linear and Non-linear Modeling software was conducted by factoring in weather variation to depict the influence of diverse environmental exposures on SB. Both weather variation and urban design played a significant role in SB. After factoring in weather variation, it was observed that children living in grid-pattern neighbourhoods closer to the city centre (with higher diversity of destinations) were less likely to be sedentary. This study demonstrates a methodology that could be replicated to integrate geography-specific weather patterns with existing cross-sectional accelerometry data to understand the influence of urban design and built environment on SB in children.

## 1. Introduction

Sedentary behaviour (SB) has emerged as an independent factor that influences a wide range of health outcomes in children [1]. Moreover, with evidence now emerging that SB embedded in childhood can continue on through adolescence into adulthood [2], it is imperative to focus on curbing SB during the early years.

Researchers have now begun focusing exclusively on SB among children [1]–[9], and investigations into the influence of environmental exposures on children's SB are gaining momentum [6]–[13]. Initial findings based on two systematic reviews suggest a stronger role of home environment, with parental support and higher socioeconomic status being associated with lower SB [3],[13]. In terms of neighbourhood environment, although perceived safety has consistently been associated with screen time [3], there is a dearth of evidence in terms of urban design and built environment's relationship with objectively measured overall SB in children.

The emphasis on environmental exposures' role in influencing SB has magnified as behavioural interventions directed at individuals have not produced a change at the population level [14]. This emphasis on environment has resulted in the gaining of prominence of an ecological research perspective (i.e., active living research) that focuses on policy driven interventions to modify urban design and built environment in the aim of facilitating increased physical activity (PA) and decreased SB at the population level [14],[15]. However, active living interventions have consistently underexplored weather variation, a phenomenon that perennially interacts with all other environmental exposures. It is especially important to factor in weather variation in temperate and continental climatic zones (Köppen-Geiger climate classification) due to a wide variation in seasonal weather in these regions [16].

Studies that have explored weather variation in populations inhabiting temperate and continental climatic zones have predominantly focused on PA and have not investigated SB exclusively. A fairly consistent finding in these studies among all age groups is that higher levels of PA are associated with higher temperatures and lower levels of PA with higher precipitation [17]–[27]. In Canada, the majority of the population experiences a wide variation in seasonal temperatures and weather conditions [28],[29]. Within Canada, prairie provinces like Saskatchewan, where this study was conducted, are known for particularly extreme variations in seasonal weather [28],[29]. While the relationship between weather variation and PA is stronger in Saskatchewan in comparison with other provinces in Canada [24], similar connection has not been established with SB.

More importantly, most studies thus far have solely focused on weather, a non-modifiable entity, instead of understanding how diverse environmental exposures influence SB after factoring in weather variation. Furthermore, in capturing weather variation, the complex and interrelated dynamics of key weather variables such as temperature, wind, and precipitation have not been taken into account. This study, by conceptualizing weather as a complex entity, accounted for weather variation in understanding the influence of urban design and built environment on objectively measured SB in children aged 10–14 years.

## 2. Materials and Methods

The study is part of an active living research initiative in Saskatoon, Saskatchewan, Canada (www.smartcitieshealthykids.com). The study protocol was approved by the University of Saskatchewan's Research Ethics Board.

### 2.1. Urban Design of Saskatoon

Presently, Saskatoon's metropolitan area population of 260,600 is spread across 65 well-defined neighbourhoods [30], where the city plays a major role in urban planning including the geographic allocation of commercial, residential and institutional land uses.

**Figure 1.**
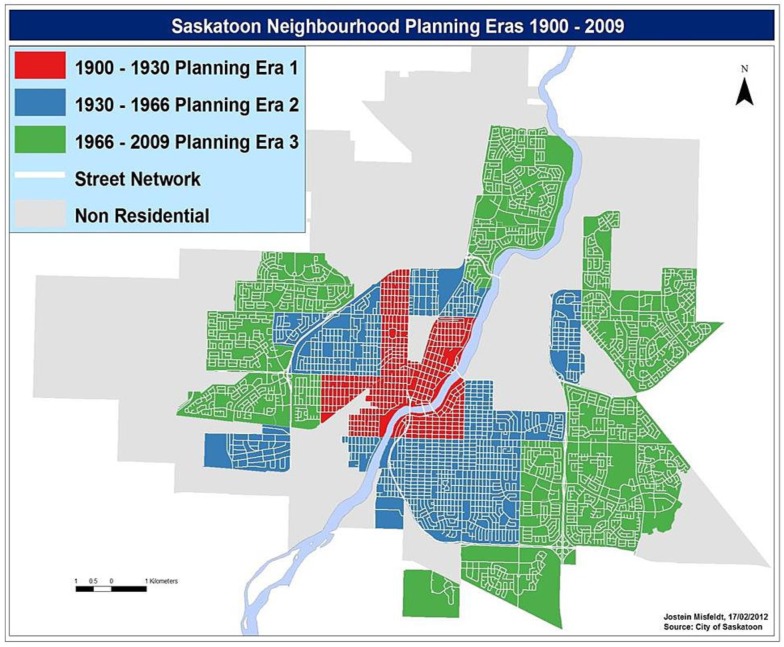
Urban design of Saskatoon depicting the three types of neighbourhoods. Red: grid; Blue: fractured grid; Green: curvilinear.

The neighbourhoods designed prior to 1930 (Planning Era 1) comprise the city centre and surrounding neighbourhoods and follow a traditional grid-patterned street design (Figure 1), typified by higher density, mixed-use neighbourhoods connected by straight, intersecting streets and back alleys. The semi-suburban neighbourhoods built between 1931 and 1966 (Planning Era 2) follow a fractured grid-pattern (Figure 1). They are predominantly residential, with lower density and become progressively car-oriented as the distance from the urban centre increases. Finally, the suburban neighbourhoods built after 1967 (Planning Era 3) follow curvilinear street patterns (Figure 1), characterized by low-density, almost exclusively residential and highly car-oriented configurations. Working with the City of Saskatoon's Neighbourhood Planning Department, our Smart Cities Healthy Kids research team has validated the three types of neighbourhoods belonging to the three different planning eras [31].

### 2.2. Neighbourhood Selection and Recruitment

The neighbourhood selection and recruitment were part of the Smart Cities Healthy Kids initiative. The sampling frame for recruiting children consisted of all 60 residential neighbourhoods in 2010 in Saskatoon categorized into the three types of neighbourhoods (Figure 1). Working with our public and Catholic school board partners, all schools in Saskatoon situated in all three types of neighbourhoods were invited to participate in the study. The recruitment was conducted through 40 out of 81 elementary schools that accepted to participate and the total study sample was representative of all 60 neighbourhoods. Working with the schools, we identified four classrooms at each elementary school (grades 5 to 8) and each school was provided with letters of consent to be given to each potential participant to deliver to their primary caregiver. In order to participate in the study, caregivers returned the signed forms to their child's homeroom teacher. It was made explicit in the consent form that caregivers or children would be able to opt out of participating at any time up until the data were pooled. Children were also provided an opportunity to decline participation even after obtaining parental consent. Of the 1610 children aged 10–14 years that agreed to participate in the Smart Cities Healthy Kids initiative from 40 schools, 455 children from 30 agreed to participate in accelerometry. This study exclusively focuses on children who participated in accelerometry.

### 2.3. Built Environment Measures

In 2009, specific built environment characteristics of all residential neighbourhoods in Saskatoon were measured utilizing two replicable tools called the Neighbourhood Active Living Potential and the Irvine-Minnesota Inventory [32],[33]. Together, these two tools were used to measure neighbourhood safety from traffic and crime, density and diversity of destinations, activity friendliness, attractiveness and pedestrian access.

### 2.4. Census-based Measures

Neighbourhood level socioeconomic variables were derived from 2006 Statistics Canada Census data and 2010 G5 Census projections to account for neighbourhood social environment [34],[35]. These included variables such as neighbourhood dwelling value, neighbourhood household income and neighbourhood unemployment rate.

### 2.5. Individual and Household Data

In 2010, prior to deploying accelerometers, Smart Cities Healthy Kids Questionnaire was administered to 455 children to capture their perception of a range of factors (household, parental, peer and neighbourhood) that influence PA. The questionnaire was pilot tested and revised as appropriate prior to field implementation. The questionnaire contained items such as: “In the last 30 days, how often have your family members provided transportation to a place where you can do PA?” and “During a typical week, how often did your friends ask you to walk or bike to school or to a friend's place?”

### 2.6. Accelerometry

Actical accelerometers (Mini Mitter Co., Inc., Bend, OR, USA) were deployed through schools to capture activity data of 455 children who completed the questionnaire. Children were visited at their respective schools and were asked to wear a belt equipped with an accelerometer around their waist to maintain proper positioning (*i.e.*, posterior to the right iliac crest of the hip) for 7 consecutive days. They were advised to remove the accelerometers during night time sleep and during any water-based activities. The devices were operationalized to measure data at 12:00 a.m. on the day following device deployment (*i.e.*, almost a full day after the device was deployed) to minimize the potential for subject reactivity within the first day of wearing the accelerometer. Accelerometers were pre-programmed to measure movement in 15-second epochs in order to capture the sporadic nature of children's activity.

The raw accelerometer data were analyzed using KineSoft version 3.3.63 (KineSoft, Loughborough, UK) to derive activity intensities using cut-points specific to the study sample's age group—SB: <100 counts/minute; light PA: 100 to <1500 counts/minute; moderate to vigorous PA: ≥1500 counts/minute [36]–[38]. The accelerometers and cut-points used in this study are the same as those used in the 2007–2009 Canadian Health Measures Survey, whose accelerometry results depicted activity patterns in a nationally representative sample of children in Canada [36]. Furthermore, using the accelerometer sample of the 2007–2009 Canadian Health Measures Survey, operational definitions and data reduction techniques were developed by Colley et al [39] Valid data for our study were derived by utilizing these population and device-specific (*i.e.*, Actical accelerometers) operational definitions and data reduction techniques, and taking into account established evidence in conducting accelerometry on large samples of children [39],[40].

Generation of valid data is essential to exclude days of accelerometry from the analysis when the participants do not wear the device for a period of time deemed sufficient to interpret levels of activity [39]. A valid day was defined as a day of accelerometry with 10 or more hours of wear-time [39]. Daily wear-time was estimated by subtracting non-wear-time of a particular accelerometry day from 24 hours. It was determined that non-wear-time would be a period of at least 60 consecutive minutes of zero accelerometer counts, including up to 2 minutes of counts between 0 and 100 [39]. The final sample consisted of children with at least four valid days including at least one valid weekend day, *i.e.*, the valid sample (N: 331; boys: 166; girls: 165 (Age 10: boys: 42; girls: 28) (Age 11: boys: 41; girls: 50) (Age 12: boys: 40; girls: 45) (Age 13: boys: 29; girls: 35) (Age 14: boys: 13; girls: 8).

However, even within valid data, there is a chance for systematic variation in daily wear-time, both within (on different days of accelerometer use) and between participants. The systemic variation occurs because even though participants are asked to wear accelerometers from the time they wake up in the morning until the time they go to bed at night, every participant wears or removes the accelerometer at her/his discretion, thus potentially introducing a random or non-random measurement bias to activity measurement. We have previously developed a methodology to control for wear-time variation and minimize measurement bias by standardization of valid data [41],[42]. The same methodology has been replicated in this study to standardize valid data.

### 2.7. Integration of Localized Weather with Cross-Sectional Accelerometry

Accelerometer data were obtained in 25 one-week cycles between April 28 and June 11, 2010 (which represented a 45 day transition period from spring to summer). Each one-week cycle of accelerometry was conducted on a different cohort of children within the total sample, with each cohort consisting of a different set of children. To match the accelerometry period, detailed weather data for the days between April 28 and June 11, 2010, were obtained from Environment Canada [43],[44].

Saskatoon experiences four distinct seasons, with average temperatures of 3.4 Celsius in spring, 17.2°C in summer, 3.2°C in autumn, and −14°C in winter. Precipitation levels are relatively low and winds usually blow from the northwest with an average speed of 15 kilometres/hour year round [45],[46]. To capture the transition from Spring to Summer, extensive exploration of the weather data was conducted to identify daily values of key weather variables corresponding to the accelerometry period: maximum temperature (Celsius), precipitation (millimetres), speed of maximum wind gust (kilometres/hour) and hours of illumination [47].

Descriptive analyses were conducted to understand the distribution (i.e., mean, median, standard deviation [SD]) of daily values of the selected weather variables during the 45 days of accelerometry. Daily values of each of these weather variables were aggregated to their corresponding one week cycle of accelerometry to calculate their mean weekly values. Thereafter, a decision rule was applied where 1SD of the distribution of daily weather values for the 45 days of accelerometry was set as the cut-point. Using this cut-point, mean weekly values for each weather variable were categorized as follows: temperature: ≥1SD = Warm, <1SD = Cold; precipitation: ≥1SD = Wet, <1SD = Dry; speed of maximum wind gust: ≥1SD = Windy, <1SD = Calm.

Finally, based on these six categories, one of the following four localized weather patterns was assigned to each week of accelerometry (weekly weather): Warm-Wet-Calm, Cold-Dry-Calm, Cold-Dry-Windy and Cold-Wet-Calm. Although, mathematically, the possible combination of weather patterns is higher than four, it is important to highlight that the classification of localized weather is based on actual weather recorded during the period of accelerometry. As the range (2.26) and SD (0.69) of hours of illumination during the 45 days of accelerometry was negligible, hours of illumination was excluded from the classification of localized weather patterns and was instead included as an independent variable in multivariable analyses.

### 2.8. Statistical Analyses

Using data from all the measures mentioned, an extensive set of predictors was derived taking into account the hierarchical nature of data distribution: neighbourhood level variables (Level 2) and individual level variables (Level 1)—Table 1. The outcome variable in all analyses was SB. Analysis of variance was conducted to assess group differences in SB between the four types of localized weather patterns (Warm-Wet-Calm, Cold-Dry-Calm, Cold-Dry-Windy and Cold-Wet-Calm), and between children residing in different types neighbourhoods. Thereafter, after factoring in weather variation, to determine the relationship between urban design and SB, fixed effects multilevel linear regression models were fitted utilizing Hierarchical Linear and Nonlinear Modeling software.

All the independent variables included in multilevel modeling were significant at the bivariate stage. Model 1 depicts the influence of localized weather patterns (with Warm-Wet-Calm category as the reference) and as well as the influence of other individual level variables. Model 2 is the final model depicting the influence of neighbourhood and individual level variables, and as well localized weather patterns. Only significant results from the final model are discussed here.

**Table 1.** Hierarchical distribution of predictors.

Hierarchy	Type of Measures	Examples of Derived Variables	Instrument
Neighbourhood Level Variables	Urban Design	Grid-Pattern	Urban Planning
		Fractured Grid Pattern	
		Curvilinear	
	Built Environment	Diversity of Destinations Density of Destinations Safety from Traffic Safety from Crime Attractiveness Pedestrian Access Universal Accessibility Activity Friendliness	Observation Tools: Neighbourhood Active Living Potential and Irvine Minnesota Inventory
	Neighbourhood Social Environment	Dwelling Value Dwellings per Acre Household Income	2006 Statistics Canada Census and G5 2010 Census Projections
Individual Level Variables	Children's Perception of Household, Neighbourhood, Peer and Parental factors	Transportation Support from Family Peer Support to Walk or Bike Household Socioeconomic Status Parents' Education	Smart Cities Healthy Kids Questionnaire
	Activity Measures	Moderate to Vigorous Physical Activity Light Physical Activity Sedentary Behaviour	Accelerometry

Note: Data obtained from built environment tools, census data and the smart cities healthy kids questionnaire were utilized to derive variables which were distributed on a numerical scale specific to each measure. Thereafter, exploration of each variable's distribution was conducted; all variables were converted into categorical variables by uniformly dichotomizing each variable's scale at the 50^th^ percentile.

## 3. Results

The study sample consisted of children aged 10–14 years residing in all three types of neighbourhoods (Figure 1: grid, fractured grid and curvilinear). Table 2 displays the three intensities of activities that represent all waking activity (moderate to vigorous PA; light PA and SB), distributed across the three types of neighbourhoods.

**Table 2.** Descriptive characteristics of the study sample depicted across urban design.

Variables	Total	Grid	Fractured Grid	Curvilinear
Sampled Schools	30	6	10	14
Total Sample	331	95	100	136
Boys	166	45	53	68
Girls	165	50	47	68
Age 10	70	16	25	29
Age 11	91	32	22	37
Age 12	85	27	26	32
Age 13	64	13	23	28
Age 14	21	7	4	10
Mean Age (SD; Min, Max)	11.6 (1.1; 10, 14)	11.6 (1.1; 10, 14)	11.5 (1.2; 10, 14)	11.6 (1.2; 10, 14)
Mean Body Mass Index (SD; Min, Max)	19.9 (4; 13.4, 35.9)	19.8 (4.2; 14, 35.9)	20.3 (4.2; 13.4, 34.3)	19.7 (3.7; 14.2, 33.8)
Mean Accelerometer Wear-time/Day (SD; Min, Max)	796.3 (51.1; 653.3, 930.2)	794.0 (53.1; 680.8, 930.2)	797.0 (53.3; 653.3, 915)	797.3 (48.1; 684.5, 910.6)
Mean MVPA/Day (SD; Min, Max)	71.2 (31.8; 8, 234.5)	72.8 (33.7; 8, 178.1)	67.3 (32.9; 13.3, 234.5)	73.1 (29.4; 16.6, 182.0)
Mean SB/Day (SD; Min, Max)	540.2 (64.8; 317.4,691.3)	537.8 (68.9; 317.4, 682.6)	546.0 (70.5; 344, 691.3)	537.3 (57.0; 379.7, 663.4)
Mean LPA/Day (SD; Min, Max)	184.7 (38.9; 92.5, 311.6)	183.3 (39.1; 104.4, 282.5)	183.0 (40.9; 92.5, 311.6)	187.0 (37.4; 98.0, 294.6)

SD: standard deviation; Min: minimum; Max: maximum; MVPA: moderate to vigorous physical activity; SB: sedentary behaviour; LPA: light physical activity. Accelerometer Wear-time, MVPA, SB and LPA values are expressed in minutes.

Children in fractured grid-pattern neighbourhoods not only accumulated more minutes of SB per day, but also fewer minutes of moderate to vigorous and light PA per day. When SB accumulation was distributed across the four types of localized weather patterns observed during data collection, children who experienced Warm-Wet-Calm weather accumulated significantly lower SB minutes per day in comparison with children who experienced all other weather patterns. Within the cold weather patterns, children who experienced Cold-Dry-Windy weather accumulated significantly higher SB per day than children who experienced Cold-Dry-Calm and Cold-Wet-Calm weather (Table 3).

**Table 3.** Group differences in sedentary behaviour between different types of localized weather patterns.

	Cold-Dry-Calm	Cold-Dry-Windy	Cold-Wet-Calm	Warm-Wet-Calm
Cold-Dry-Calm	0.00			
				
Cold-Dry-Windy	9.96**	0.00		
				
Cold-Wet-Calm	−2.69	−12.66 ***	0.00	
				
Warm-Wet-Calm	−11.06***	−21.03 ***	−8.36 **	0.00

Note: Each value presented in the tables is a result of subtraction of SB between 2 types of localized weather patterns (values in rows subtracted from values in columns); *** *p* < 0.001; ** *p* < 0.01.

When analysis of variance was conducted to observe the distribution of SB across different types of neighbourhoods during all types of localized weather patterns, a consistent pattern emerged (Table 4). During all weather patterns, children residing in fractured grid-pattern neighbourhoods accumulated significantly more SB per day in comparison with children residing in other types of neighbourhoods. The accumulation of SB was particularly higher during Warm-Wet-Calm weather, where children in fractured grid-pattern neighbourhoods accumulated 20.01 and 13.70 more minutes of SB per day in comparison with children in curvilinear and grid-pattern neighbourhoods, respectively (Table 4).

**Table 4.** ANOVA testing group differences in sedentary behaviour between different types of neighbourhoods stratified by localized weather patterns.

SB accumulation		Grid	Fractured	Curvilinear
Warm-Wet-Calm	Grid	0.00	−13.7*	6.31
	Fractured	13.7*	0.00	20.01***
	Curvilinear	6.31	−20.01***	0.00
Cold-Dry-Windy	Grid	0.00	0.00	N/A
	Fractured	0.00	0.00	N/A
	Curvilinear	N/A	N/A	0.00
Cold-Dry-Calm	Grid	0.00	−7.24	4.29
	Fractured	7.24	0.00	11.53***
	Curvilinear	−4.29	−11.53***	0.00
Cold-Wet-Calm	Grid	0.00	−9.19***	−4.09
	Fractured	9.19***	0.00	5.10
	Curvilinear	4.09	−5.10	0.00

Note: Each value presented in the table is a result of subtraction of group SB between 2 types of urban design values in rows subtracted from values in columns); *** *p* < 0.001; ** *p* < 0.01; * *p* < 0.05 fractured grid's detrimental effect has been highlighted in bold; ANOVA: analysis of variance; SB: sedentary behaviour.

Multilevel multivariable modeling reiterated the influence of localized weather patterns on SB accumulation in children (Table 5). In comparison with children who experienced Warm-Wet-Calm weather, children who experienced Cold-Dry-Calm (odds ratio [OR] = 39.50; 95% confidence interval [CI] = 2.21–70.83) and Cold-Dry-Windy weather (OR = 42.45; CI = 1.66–108.25) were more likely to accumulate higher SB. Moreover, children who experienced greater hours of illumination per day were less likely to accumulate higher SB (OR = 0.81; CI = 0.68–0.98).

In terms of urban design of Saskatoon, even though descriptive analyses and analysis of variance showed that children residing in fractured grid-pattern neighbourhoods consistently accumulated more SB in comparison with children living in other neighbourhoods, this finding was not observed in multilevel models. With respect to age, children aged 13 years were more likely to accumulate higher SB than children aged 10 years (OR = 7.75; CI = 2.14–28.01).

**Table 5.** Multilevel linear regression model predicting the influence of localized weather patterns and urban design on sedentary behaviour.

Variables	Null Model		Model 1		Model 2	
	OR	CI	OR	CI	OR	CI
Intercept	2.16	1.45–3.10	1.26	0.72–3.23	0.14	0.00–0.89
Mean Hours of Illumination			0.72 **	0.29–0.82	0.81 **	0.01–0.83
Cold-Dry-Windy vs. Warm-Wet-Calm			37.82 *	2.38–104.67	42.50 *	1.66–108.25
Cold-Dry-Calm vs. Warm-Wet-Calm			34.63 *	4.58–73.85	39.50 *	2.21–70.83
Cold-Wet-Calm vs. Warm-Wet-Calm			2.41	1.14–16.59	3.41	0.86–13.48
Boys vs, Girls			0.96	0.52–2.54	0.98	0.53–1.80
Age 11 vs. Age 10			2.39	040–6.42	1.82	0.83–4.01
Age 12 vs. Age 10			2.27	0.38–3.72	1.68	0.77–3.67
Age 13 vs. Age 10			3.11 *	1.12–28.30	7.75 *	2.14–28.01
Age 14 vs. Age 10			6.20	0.23–60.02	7.75	0.95–65.80
Fractured Grid vs. Grid					0.77	0.34–1.74
Curvilinear vs. Grid					1.32	0.61–2.88

OR: odds ratio; CI: confidence interval; SB: sedentary behaviour; * *p* < 0.05; ** *p* < 0.01; *** *p* < 0.001; Model 1 depicts the influence of localized weather patterns (with Warm-Wet-Calm category as the reference) and as well as the influence of other individual level variables. Model 2 is the final model depicting the influence of neighbourhood and individual level variables, and as well localized weather patterns.

## 4. Discussion

The purpose of this study is to understand how urban design and built environment influence children's SB after taking into account weather variation. This approach to understand patterns of SB is essential because weather is a perennial factor that interacts with all other environmental variables.

Moreover, as weather is a complex entity consisting of several interrelated elements [48], localized weather patterns were derived and integrated with cross-sectional accelerometry. Integration of cross-sectional accelerometry with localized weather patterns depicted that weather variation influences children's SB even in a single seasonal transition (spring to summer).

Warm-Wet-Calm weather was consistently associated with lower SB accumulation and Cold-Dry-Windy weather was associated with higher SB accumulation. It is apparent that exposure to higher daily temperatures played a role in lower SB accumulation. Although previous studies have shown that higher temperatures in temperate climatic zones are associated with higher PA [17]–[27], similar findings have not been established for SB. Beyond temperature, wind speed played a significant factor in SB accumulation, with lower wind speeds being associated with lower SB and greater wind speeds being associated with higher SB. These observations not only underline the complexity of weather, but also the need to account for the interrelatedness and dynamics of specific characteristics such as temperature, wind speed and precipitation [47]. However, the results also showed ambiguity because cold-dry-calm weather, which depicts lower wind speed, was also associated with higher SB. Further research with longitudinal data is required to understand these complex patterns.

Nevertheless, as weather is non-modifiable, our ultimate focus was on urban design and neighbourhood built environment. After factoring in weather variation, a pattern emerged when analysis of variance was conducted to assess group differences in SB between children residing in different types neighbourhoods. Children residing in neighbourhoods with fractured grid-pattern neighbourhoods consistently accumulated more SB in comparison with children residing in neighbourhoods with grid-pattern and curvilinear urban design.

There are several explanations for this pattern. The grid-pattern neighbourhoods surrounding the city centre (Figure 1: Planning Era 1), by virtue of their mixed land-use (combination of commercial, residential, institutional establishments), possess greater density and diversity of destinations, are less car-oriented, and more pedestrian friendly [31]. The usage of the term ‘grid-pattern’ is due to the structure of road networks in these neighbourhoods that have multiple intersections and interconnected streets, which ultimately provide greater access to the many destinations available due to mixed land-use. While mixed land-use is known to be a strong predictor of PA among adolescents [49], SB's relationship with mixed land-use in children has not been established. Thus, the finding that children living in mixed land-use (i.e., grid-pattern) neighbourhoods accumulated lower SB indicates that mixed-land use neighbourhoods are beneficial for overall active living in children (i.e., higher PA and lower SB).

Children residing in curvilinear neighbourhoods (Figure 1: Planning Era 3) that are characterized by low-density, almost exclusively residential and highly car-oriented configurations [31], also accumulated lower SB than children residing in fractured grid-pattern neighbourhoods. These neighbourhoods represent the higher socioeconomic areas of Saskatoon and existing evidence indicates that higher socioeconomic status and parental support are associated with lower SB in children [3],[13]. Curvilinear urban design features are different in comparison with grid-pattern features, yet the association of two dissimilar types of urban design with lower SB shows that active living communities could be created through the adoption of selective features from different types of urban design.

### Strengths and Limitations

The primary strength of the study is that it addresses a key gap in evidence in active living research by factoring in weather variation to understand the influence of urban design and built environment on objectively measured SB in children. Another strength of the study is the integration of localized weather patterns with cross-sectional accelerometry, a methodology which allowed the capture of weather variation in a single seasonal transition.

However, there are several limitations. Even though objective SB data were derived from accelerometers, the lack of social and spatial context related to SB accumulation poses difficulty in establishing accurate understanding of *how* SB was accumulated within different environmental contexts or *where* (neighbourhood, indoor/outdoor, playground, recreational facility, etc.) SB was accumulated. Objective SB that was derived from accelerometry was an overall estimation of sedentariness and data regarding the type of SB, especially screen time was not collected. Studies are now emerging which utilize ecological momentary assessments and global positioning systems that would allow the capture of different types of SB within overlapping social and physical contexts [50],[51]. These advances, when combined with accelerometry, would provide the methodological depth to tease out the complex pathways that determine SB accumulation.

Finally, the study is limited by cross-sectional data which, when analyzed using multilevel modeling, did not depict a significant relationship between urban design and SB. Nevertheless, after factoring in weather, the consistent pattern of urban design's influence on SB depicted through analysis of variance needs to be corroborated with longitudinal studies that investigate the relationship between urban design and SB in all seasons.

## 5. Conclusion

It is imperative to understand how urban design and built environment influence SB in children after taking weather variation into consideration. This study provides a methodology to leverage cross-sectional accelerometry to capture weather variation in all jurisdictions to understand urban design and built environment's influence on SB. Moreover, this methodology could be replicated with longitudinal data to confirm the results depicted in this study. The evidence thus generated is useful to inform urban planning policy across jurisdictions to promote neighbourhood development that facilitates not only an increase in PA, but also a decrease in SB in children through all seasons.
